# Renal hydatid cyst; a rare infectious disease

**DOI:** 10.1093/omcr/omz011

**Published:** 2019-05-03

**Authors:** Hafezi Ahmadi Mohammad Reza, Gheitasi Rreza, Barati Nastaran, MotavalliHaghi Mousa

**Affiliations:** 1 Department of Pathology, Ilam University of Medical Sciences, Ilam, Iran; 2 Biotechnology and Medical Plants Research Center, Ilam University of Medical Sciences, Ilam, Iran; 3 Department of Immunology, School of Medicine, Hamadan University of Medical Sciences, Hamadan, Iran; 4 Vice-Chancellor for Research and Technology, Hamadan University of Medical Sciences, Hamadan, Iran; 5 Department of Medical Parasitology and Mycology, School of Medicine, Hamadan University of Medical Sciences, Hamadan, Iran

## Abstract

Hydatid cyst is one of the most important zoonotic diseases that is caused by *Echinococcus granulosus*. Infection is transferred through the oral-fecal pathway by eggs of the parasite and more by eating vegetables and food contaminated with dog stool containing eggs of the parasite. Hydatid cyst can be made in liver, lung and rarely in heart, breast, thyroid, soft tissue of neck and kidney. Hydatid cyst of the kidney is a rare disease which may have no symptoms for years. In this case report, the patient has had a typical cyst in the left kidney for years that it seems to be silent for long time in a woman with ambiguous left flank pain whom went to a physician, and after complementary examinations, finally a typical hydatid cyst was diagnosed with the size of 93 × 120 mm in left kidney.

## INTRODUCTION

Hydatid cyst is one of the most important zoonotic diseases made by Cestoda *Echinococcus*, especially *Echinococcus granulosus*. The final hosts of the parasite are dogs and Canidae and the intermediate hosts are the livestock such as cows, sheep and goats. The infection is caused by oral-fecal route and transferred by parasite eggs, mostly due to consuming infected vegetables and food by stool of dog containing the parasite egg. The eggs are splitted in the intestinal duct; the larva enters to the blood through the intestinal mucosa. The disease is endemic in different parts of the world in which animal husbandry is common such as India, south of America, the Middle East, Mediterranean Australia and New Zealand [1–4]. Larvae of the parasite in intermediate hosts is capable to creae cysts in all body organs. Liver (50–70%) and lungs (20–30%) are mostly involved organs in hydatid cyst, but it is rarely seen in other organs like heart, breast, thyroid, the soft tissue of neck and kidney [5]. Hydatid disease is defined by the existence of hydatid cyst in human which may be without any symptoms for a long time [6]. The symptoms are seen when the cyst is enlarged in the involved organs and also is identified accidentally in operation and paraclinical tests such as pathologic imaging or performing serologic tests. Operation and using puncture, aspiration injection and re-aspiration (PAIR) are the most important and best treatment methods. According to the pressure on hydatid cyst, getting torn and the release of hydatid liquid containing protoscolex during the surgery are not unusual, and this issue is the most important cause of invasion in this disease, therefore applying scolicidals during the surgery is necessary.

## CASE HISTORY

**Figure 1:**
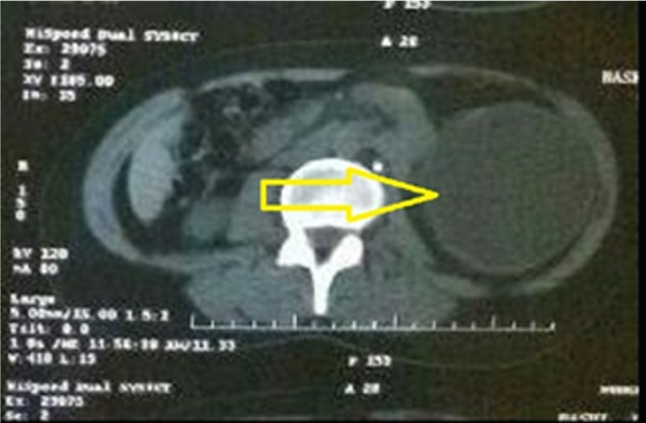
CT scan for kidneys. Right kidney with a normal dimension of 23×121 mm and without stone or obstruction. Left kidney with a dimension of 18×165 mm and the existence of a typical cyst with a dimension of 93×120 mm.

**Figure 2:**
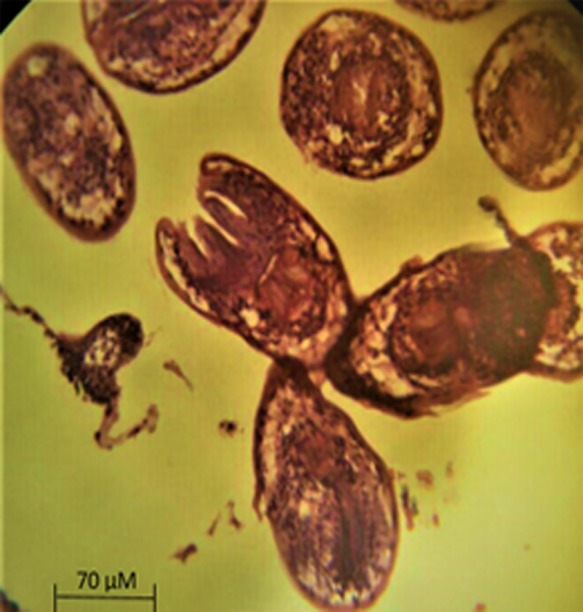
Tissue section and protoscoleces of hydatid cyst (×1000 magnification)

A 26-year-old woman, resident of Mehdi Abad (Ilam province) with vague abdominal pain, was referred to urology department. In physical examination, low blood pressure and pervasive tenderness with the priority of left lower quadrant of the abdomen were found, and other vital signs were normal, and the patient did not have any apparent history. With regard to the symptoms and the involved organs, general tests such as the analysis of blood and biochemistry factors and also the urine analysis were prescribed for the patient. The analysis of blood and biochemistry factors was normal, but in urine analysis, a little mucus, epithelial cell and a high amount of RBC were seen and then sonography of kidneys was prescribed for the patient. Sonography of right kidney showed the normal dimension of 121 mm and parenchyma thickness of 23 mm, without any stone or obstruction but in left kidney with a dimension of 165 mm and parenchyma thickness of 18 mm, one typical cyst with a dimension of 120 × 93 mm was seen. Computed tomography scan illustrated the cyst in details (Fig. 1). Result of ultrasound and CT were showed a simple cyst for R/O cancer and without any clear diagnosis, cystectomy was scheduled for the patient, and the specimen was sent to the pathology department. Results showed the definitive diagnosis of hydatid cyst and the protoscoleces were seen in tissue section (Fig. 2). After the surgery, the patient was discharged from the hospital without any symptoms. After diagnosis of pathologist, the patient received Albendazole 10 mg/kg daily for 12 weeks. Patient was followed up for 3 months, every 2 weeks by ultrasound, liver test, urine analysis, CBC and platelet count test to look for any cysts. Afterwards, the patient was followed up monthly for 1 year, and no recurrence was observed.

## DISCUSSION

Echinococcosis is a zoonotic disease and created by the larva of *Echinococcus granulosus* parasite. In the life cycle of this parasite, dogs are the main hosts of *Echinococcus* mature worm, also vegetarians are considered as intermediate host of this parasite, so the humans may be intermediate host by swallowing eggs containing larva of *Echinococcus granulosus* due to touching dogs or consumption of contaminated vegetables and water. When the embryonic membrane gets torn, firstly larva enters the abdominal area through penetrating the intestinal walls, then gets into the intermediate portal vein system or lymphatic system and reaches to different organs. The reaction of parasites against the immunity system is too complex and has been vague yet. Despite the existence of inherent and acquired immunity system, host and parasites interact with each other. *Echinococcus granulosus*’s larva can tolerate against anti-parasite responses of host by creation of laminated layer, versus, host can prevent from hydatid cyst development by creation of fibrotic layer around it. However in many cases, it remains hidden and can be enlarged depending on its position for a long time and finally hydatid cyst may detect by symptoms in the patient such as damaging or putting pressure on the involved organ and marginal organs or patient may see a doctor for other challenge of body and hydatid cyst is detected accidentally. The mostly involved organs in human hydatid cysts are liver (about 75 %), lung (15%) and other organs are less involved [7, 8]. Renal hydatid cyst is the third rank (2–4%) which is a rare and important disease. Hydatid cyst is an important health problem in the world, especially in endemic regions, it is widespread more than 5 to 10% in parts of Argentina, Peru, East Africa, central Asia and China [9]. Renal hydatid cysts may be silent for years and do not show any obvious symptoms. In this case report, the patient had a hydatid cyst in kidney for years, and because it did not show specific symptoms, it was silent and progressive. This silence causes irreparable damage to the involved organs, and unfortunately, the patient may lose the involved organ. Combination of diagnostic methods is necessary for diagnosis of hydatid cyst in some organs with no symptoms, like kidney, such as clinical history, serological analysis, urine analysis and medical imaging that finally confirmed the disease by showing hydatid cyst,layers of hydatid cyst or protoscolex.For treatment of these cases both pharmacotherapy and surgery are needed. For kidney hydatid cysts, PAIR (puncture- aspiration- injection- re-aspiration) is a successful method of therapy, although when the cysts are larger than 6 cm, embedding the subcutaneous tube and evacuating the contents of the cyst are needed several times to decrease the risk of infection because the fluid of hydatid cyst is containing protoscolex. The best method of therapy is surgery and removing the hydatid cyst of the kidney without getting torn, and it depends upon the status and size of the cyst. Moreover, scolicidal must be taken before the surgery to decrease the risk of infection.

## CONCLUSIONS

According to this study and similar studies, it seems that diagnosis of hydatid cyst of the kidney, needs combination of methods such as laparoscopy, imaging, disease history, serology, hematology, and urinary analysis. Of course for removing the cyst scolicidal drugs must be used before surgery, and so involved organ, status and size of the hydatid cyst should be regarded to minimize the possibility of hydatid cyst tearing and consequently double pollution in other organs.
